# Online flow cytometry reveals microbial dynamics influenced by concurrent natural and operational events in groundwater used for drinking water treatment

**DOI:** 10.1038/srep38462

**Published:** 2016-12-07

**Authors:** Michael D. Besmer, Jannis Epting, Rebecca M. Page, Jürg A. Sigrist, Peter Huggenberger, Frederik Hammes

**Affiliations:** 1 Department of Environmental Microbiology, Eawag, Swiss Federal Institute of Aquatic Science and Technology, Dübendorf, Switzerland; 2 Department of Environmental Systems Science, Institute of Biogeochemistry and Pollutant Dynamics, ETH Zürich, Zürich, Switzerland; 3 Applied and Environmental Geology, Department of Environmental Sciences, University of Basel, Basel, Switzerland; 4 Endress+Hauser (Schweiz) AG, Kägenstrasse 2, 4153 Reinach, Switzerland

## Abstract

Detailed measurements of physical, chemical and biological dynamics in groundwater are key to understanding the important processes in place and their influence on water quality – particularly when used for drinking water. Measuring temporal bacterial dynamics at high frequency is challenging due to the limitations in automation of sampling and detection of the conventional, cultivation-based microbial methods. In this study, fully automated online flow cytometry was applied in a groundwater system for the first time in order to monitor microbial dynamics in a groundwater extraction well. Measurements of bacterial concentrations every 15 minutes during 14 days revealed both aperiodic and periodic dynamics that could not be detected previously, resulting in total cell concentration (TCC) fluctuations between 120 and 280 cells μL^−1^. The aperiodic dynamic was linked to river water contamination following precipitation events, while the (diurnal) periodic dynamic was attributed to changes in hydrological conditions as a consequence of intermittent groundwater extraction. Based on the high number of measurements, the two patterns could be disentangled and quantified separately. This study i) increases the understanding of system performance, ii) helps to optimize monitoring strategies, and iii) opens the possibility for more sophisticated (quantitative) microbial risk assessment of drinking water treatment systems.

Groundwater is a vital source of drinking water worldwide^1^,^2^. Natural processes influence water quality in groundwater aquifers through the removal of nutrients, pollutants, and microorganisms as well as the addition of minerals^3^,^4^. Many of the abiotic processes and dynamics in aquifers are relatively well understood due to suitable monitoring and modeling tools^5^,^6^,^7^. In contrast, knowledge on microbial dynamics is considerably more limited and studies have thus far focused mainly on the filtration efficacy of river banks and aquifers with respect to indicator organisms^8^,^9^,^10^, source tracking of pathogens from point sources^11^,^12^,^13^, characterization of microbial communities^14^,^15^,^16^,^17^, and nutrient removal through biofilms^18^,^19^,^20^. While these studies yielded valuable information on the influence of aquifers on microbial water quality, only little is known about the processes and dynamics of short-term changes in microbial concentrations and composition in groundwater^21^. Ignorance of the existence and characteristics of such microbial dynamics may well lead to incorrect sampling/monitoring strategies and an erroneous assessment of water quality^22^.

Both natural and man-made temporal dynamics in chemical and microbiological variables can be expected in groundwater. Driving forces for these include: i) seasonal variations (e.g., temperature, precipitation, vegetation, land-use, infiltration volumes and pathways), ii) natural events (river floods, rainfall, snowmelt), and iii) water extraction (in various technical configurations)^21^,^23^. For example, groundwater wells in close proximity to river water are particularly vulnerable to fluctuations in the river water quality^24^. In this context, the short-term dynamics (hours-to-weeks) of natural and especially operational events have so far mainly been looked at with respect to hydrology and abiotic water quality, but only scarcely from the perspective of microbial water quality^5^,^25^,^26^,^27^. Monitoring the microbial quality of extracted groundwater for drinking water is typically confined to the conventional approach of infrequent grab sampling and conventional cultivation-based detection methodology^28^. The costs and labor-intensive nature of the latter hinder the investigation of short-term dynamics at adequate temporal resolution. While infrequent measurements of raw and treated water can capture long-term (months-to-years) trends such as seasonal changes in abiotic and microbial variables^29^,^30^,^31^, it fails to capture dynamics on shorter terms (hours-to-weeks)^32^,^33^. Specifically, with infrequent sampling a considerable amount of uncertainty remains with respect to i) the predominant contaminant concentration levels (e.g., prevalent dry-weather conditions in temperate climates), ii) the frequency of deviations from this level, iii) whether the deviations are real (rather than single erroneous measurements), and iv) the full magnitude and duration of the deviations.

High frequency (online) microbial monitoring of environmental samples is becoming increasingly feasible with the emergence of new microbial measurement tools^34^,^35^,^36^,^37^. In this study, fully automated online flow cytometry^34^ was employed to monitor a riverbank filtration drinking water treatment system at high frequency for two weeks. The goal was to assess the value of online microbial monitoring for quantifying and characterizing dynamic fluctuations in bacterial concentrations in groundwater. The novelty of this work is i) the *in-situ* application of online flow cytometry for high frequency microbial monitoring of an actual drinking water treatment system, and ii) the elucidation of both natural and man-made influences on microbial water quality.

## Materials and Methods

### Study site

**Figure 1:**
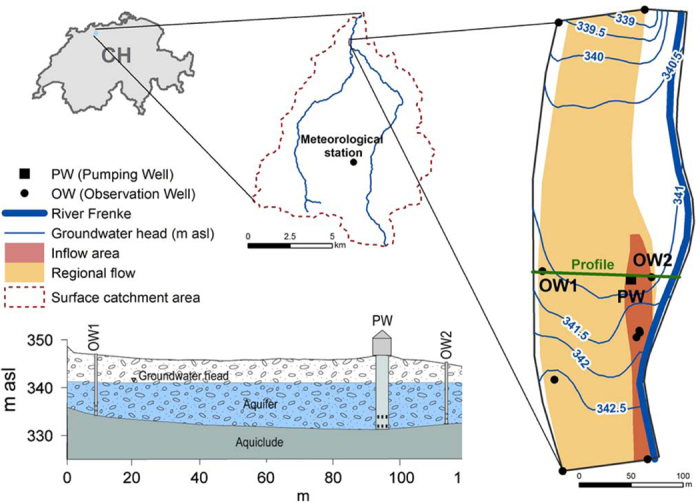
Overview and cross section of the study area showing the monitored extraction well (PW) and the two observation wells (OW1 and OW2). The modeled regional groundwater flow is indicated in yellow color and the local infiltration from the river is indicated in red color. The figure was created using Esri ArcMap 10.1 (http://desktop.arcgis.com/en/arcmap/), GMS 10.0.11 (http://www.aquaveo.com/software/gms-groundwater-modeling-system-introduction) and Adobe Illustrator CS4 (http://www.adobe.com/products/illustrator.html).

The study area is located in the lower Frenke Valley in Northwestern Switzerland (Fig. 1). The alluvial aquifer of the Quaternary valley fill consists of sandy, variable sorted carbonate gravel (Triassic and Jurassic rock components) with intercalations of silt and clay layers of variable extent. This results in a range of hydraulic properties. The Frenke River was canalized at the end of the 19^th^ century and the riverbed was disconnected and lowered several meters below the former floodplain. The geological units of the surrounding hills and the bedrock below the gravelly valley fill consist of Jurassic carbonate formations (Hauptrogenstein) with variable karstification. In addition, large-scale analysis of tectonic structures (Horst and Graben structures of the Tabular Jura) and the results of hydrogeophysical investigations (electrical resistivity tomography) indicate the existence of several fault systems. Thus, beside the groundwater recharge by infiltrating river water, both the interaction with the regional groundwater flow, and the regional Karst, respectively fault system have to be considered. Figure 1 shows the location of the drinking water extraction well (PW) approximately 35 m west of the river, as well as the groundwater observation wells to the east (OW2) and the west (OW1) of the extraction well forming a transect. The water in the extraction well had the following composition: pH 7.5–7.7, electrical conductivity 0.65–0.68 mS cm^−1^, dissolved oxygen 3–5 mg l^−1^, temperature 14.1–14.4 °C. The drinking water treatment plant intermittently extracts groundwater from a single well, which is then treated with UV-disinfection prior to storage and distribution.

### Online flow cytometry

A continuously flowing sampling line that operated independently of the drinking water extraction pumps was installed in the extraction well. Samples were drawn from this sampling line every 15 minutes during 14 days, equaling 1′314 individual samples. An automated staining module was combined with an Accuri C6 flow cytometer (BD Accuri, San Jose CA, USA) as described previously^34^. In short, water samples were drawn every 15 minutes and mixed with fluorescent stain (SYBR Green I [Life Technologies, Eugene OR, USA], final concentration 1:10′000). After incubation (10 min; 37 °C), the sample was transferred to the flow cytometer and measured at a flow rate of 66 μL min^−1^ for 90 s with a lower threshold on the green fluorescence (FL1-H) set at 1′000 (for further details on the flow cytometer setup see Prest *et al*.^38^). The staining module was rinsed with nanopure (deionized, 0.22 μm filtered) water after each measurement and extended cleaning with hypochlorite and detergent was performed daily. Flow cytometry data was exported as fcs files and batch processed with custom software^34^. In short, fixed gates^38^ were used to separate bacteria and background signals and additionally to distinguish between so-called high (HNA) and low (LNA) nucleic acid content bacteria.

### Meteorological and hydrological measurements and operational information

Precipitation data were collected as 30 min cumulative values at a meteorological station located approximately 7.5 km south of the extraction well in the catchment area of the river (Fig. 1). The river water level was recorded with a STS DL/N 70 data logger approximately 100 m upstream of the extraction well (Fig. 1). The groundwater levels in the two observation wells were continuously recorded with an OTT Orpheus Mini data logger. Water extraction intervals were obtained directly from the water utility’s control system. Extraction primarily occurred at night due to lower electricity prices. Two identical pumps with a maximum extraction rate of 146 m^3^ h^−1^ are operated alternately to avoid overheating.

### Time series decomposition

Online flow cytometry measurements were linearly interpolated and then sampled at equal time intervals (15 minutes) using the “approx()” command of the statistical software R^39^. This was to adjust for minor deviations from the 15 minutes sampling interval in the original data set (e.g., due to maintenance on the measurement system). The interpolated time series was then decomposed into a “trend component” (aperiodic dynamic), a “seasonality component” (periodic dynamic; R-code based terminology not related to seasons of the year), and a “remainder component” (noise and dynamics not accounted for by the above two components) using the “stl()” command^39^,^40^ with “s.window” set to “periodic” (R-code based terminology not related to the observed periodic dynamic) and all other parameters set to default. The “trend component” and the “seasonality component” were then added up manually and plotted.

## Results

### Precipitation events and river stage fluctuations

**Figure 2:**
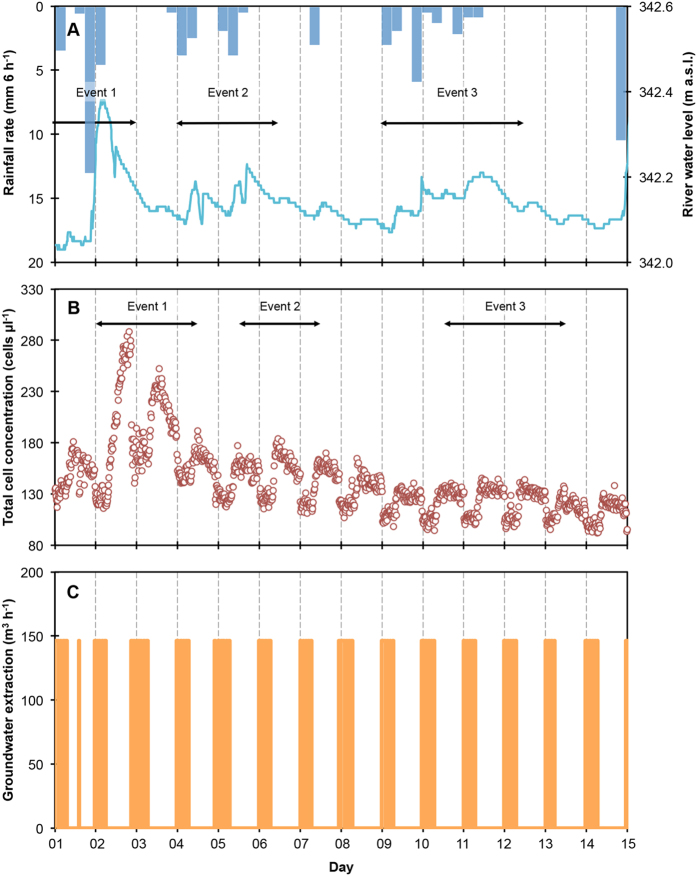
Precipitation rate (**A**) (dark blue bars), river water level (**A**) (light blue), flow cytometry measurements of TCC (n = 1′314) (**B**) (red circles) in groundwater in the extraction well, and groundwater extraction rate (**C**) (orange). The groundwater at the extraction well is influenced by both riverbank filtrate and the regional groundwater (Fig. 1). Approximate event periods are indicated for easier reference in the text.

Precipitation data show three substantial events (here defined as a period with more than 10 mm total precipitation and less than 24 h without precipitation) within the 14-day observation period (Fig. 2A). The first event was approximately 22 mm in 48 h (days 1 and 2), and resulted in the most prominent river stage increase (±0.35 m). The second event was approximately 13 mm in 60 h (days 4–6, noon) and the third event was approximately 16 mm in 84 h (days 9–12, noon). The river stage increase was considerably less during the second and third events compared to the first one. The river stage did not return to initial values of 342.0 meters above sea level (m a.s.l.) (day 1) during the entire observation period.

### Microbial dynamics

Dry weather TCC measurements collected prior to this campaign showed the river water containing 629 ± 34 cells μL^−1^ (n = 15) and the extraction well approximately 10-fold less with 73 ± 11 cells μL^−1^ (n = 47) and suggested a diurnal pattern in the extraction well data (Figure S1, Supplementary information). In the present sampling campaign, automated flow cytometry TCC measurements of the water in the extraction well at 15-minute intervals during two weeks were noticeably higher (118–288 cells μL^−1^; n = 1′314) compared to the dry weather data. Moreover, the data revealed a periodic dynamic (i.e. recurring at uniform intervals and magnitudes) as well as an aperiodic dynamic (i.e. recurring at non-uniform intervals and magnitudes) (Fig. 2B).

The periodic TCC dynamic manifested in a diurnal pattern with two discernable stages: high TCC concentrations during the day (ca. 08:00–24:00) and low TCC concentrations during the night (ca. 00:00–08:00) (Fig. 2B; Figure S1, Figure S2). The transition between high TCC and low TCC periods was fast (i.e. 1–2 hours) and the difference between daily maxima and minima was usually around 40 cells μL^−1^ (ca. 25%), but increased during Event 1 (see below). The diurnal fluctuations in TCC corresponded with the operation of the extraction pumps in the treatment plant (Fig. 2C). Water extraction was intermittent throughout the experimental period and occurred on a daily basis during the night (mostly between 23:00 and 08:00). When operating, the pumps ran at full capacity (146 m^3^ h^−1^), and extraction periods coincided with the lowest TCC concentrations observed in the extraction well (Fig. 2B and C; Figure S2).

An aperiodic TCC dynamic was recorded concurrent with the diurnal TCC pattern and was considerably more apparent during non-extraction (high TCC) periods (Fig. 2B; Table S1). TCC values started around 130 cells μL^−1^ (day 1), increased rapidly and peaked on day 2 (maximum = 288 cells μL^−1^, Event 1) before decreasing to a daily maximum value of 165 μL^−1^ on day 5. A minor TCC increase was observed on day 6 (maximum value = 183 cells μL^−1^, Event 2), followed by a further decrease (maximum value = 126 cells μL^−1^; day 9). A second minor increase in TCC was observed on days 10–12 (maximum value = 145 cells μL^−1^, Event 3), followed by a final decrease on days 13 and 14 (maximum value = 118 cells μL^−1^). For a further overview of the TCC fluctuations, Table S1 provides moving average minima (during extraction) and maxima (during periods with no extraction) values of TCC for each day. In contrast to the first event, the later two precipitation-induced high-discharge events did not cause sharp peaks in TCC, but arguably prevented it from decreasing and reaching dry-weather conditions (minimum: 54 cells μL^−1^; maximum: 93 cells μL^−1^; average: 73 cells μL^−1^; Figure S1). The aperiodic TCC fluctuations (Fig. 2B) are attributed to the influence of precipitation events on the river water (Fig. 2A), with each TCC event occurring approximately 15 h after the corresponding increase in the river water stage.

**Figure 3:**
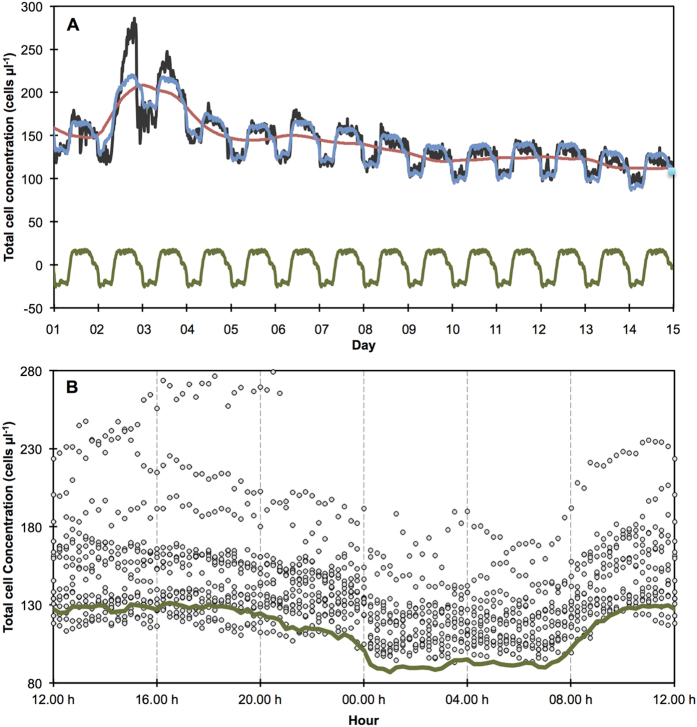
Decomposition of online flow cytometry based TCC values (**A**) depicted in Fig. 2B. From the original time series (black line) the “trend component” (aperiodic dynamic, red line) and the “seasonality component” (periodic dynamic, green line) were extracted. The sum of these two components (blue line) explains the measured values to a good degree with some larger deviation during Event 1 (days 2–3). Comparison of all TCC values from Fig. 2B (black circles) against the established dynamic baseline (i.e. periodic dynamic from panel A) (green line) (**B**). For easier comparison, the extracted periodic fluctuation (i.e. “seasonality component” from time series decomposition was scaled to the lowest trend value (day 15).

In order to better elucidate and characterize the periodic and aperiodic dynamics, we applied a time series decomposition. This method basically iteratively extracted a mean “seasonality component” (i.e. periodic dynamic) and a smoothed “trend component” (i.e. aperiodic dynamic) from the original TCC time series (Fig. 2B) (see materials and method section for details). The results in Fig. 3 illustrate the different characteristics of the two concurrent dynamics. The superposition of the “trend component” and the “seasonality component” explained most of the observed pattern in the TCC time series. However, particularly during Event 1 it did not explain the full magnitude of the observed fluctuations. It is also noticeable that the influence of the precipitation event on the periodic diurnal pattern was much more pronounced during non-extraction conditions than during extraction conditions. For example, the absolute difference between TCC values in the extraction and non-extraction period more than tripled during Event 1 on days 2 and 3 compared to the difference during most of the other diurnal fluctuations. This implies that additional factors/effects influenced the system significantly during this period (discussed below).

**Figure 4:**
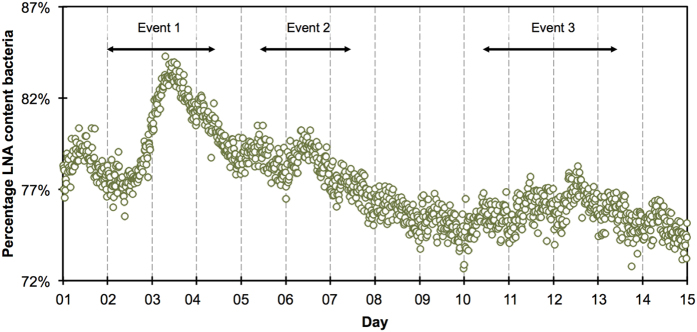
Flow cytometry measurements (n = 1′314) of the percentage of low nucleic acid (LNA) content bacteria (green circles) in groundwater in the extraction well. Approximate event periods are indicated for easier reference in the text.

Changes in the flow-cytometric fluorescence fingerprints, in this study presented as the percentage of so-called low-nucleic acid (LNA) content bacteria, largely corroborated the changes in TCC fluctuations (Fig. 4). The aperiodic dynamic started around 78% LNA content bacteria (days 1 & 2), and peaked at 84% (Event 1, day 3) before dropping to 79% (days 4–6). After the peak of Event 2 (day 6) around 80%, it slowly decreased to 75% (days 7–9) before rising to 77% during Event 3 (days 10 to 12) and eventually slowly decreasing to 74% (days 13 to 14). Interesting observations are that during Event 1 (days 2–3) the percentage of LNA content bacteria peaked almost 24 hours after TCC, while Event 3 and Event 4 were more evident in the percentage of LNA content bacteria compared to TCC. On the other hand, the periodic diurnal patterns were less distinct with respect to the percentage LNA content bacteria, with about 2 percentage-points lower values during extraction periods compared to non-extraction periods.

### Hydrological dynamics influenced by precipitation events and water extraction

**Figure 5:**
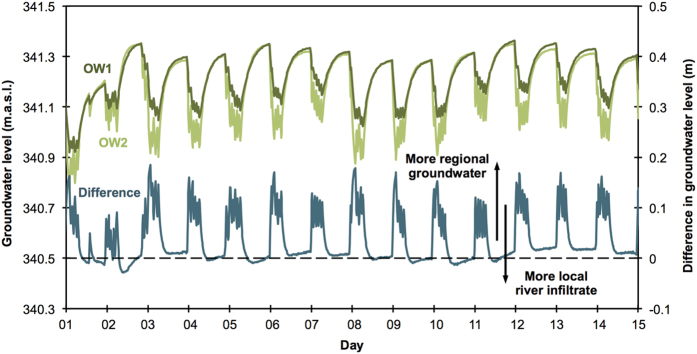
Groundwater levels in observation wells OW1 (dark green, left axis) and OW2 (light green, left axis) and the difference in groundwater levels between OW2 and OW1 (turquoise, right axis). The black arrows indicate the influence of the difference in groundwater levels on the ratio of the two groundwater types in the extraction well.

Water levels in the two observation wells (OW1 and OW2) on either side of the extraction well (Fig. 1) fluctuated between 340.8 and 341.4 m.a.s.l. (Fig. 5) and showed similar temporal dynamics as TCC (Fig. 2B). A periodic, diurnal pattern was observed in both data series with daily variations of around 0.4 m and this pattern corresponded clearly with the water extraction (Fig. 2C) and the TCC fluctuations (Fig. 2B). A rapid drop in water levels occurred shortly before midnight and water levels started to increase again around 09:00 to reach a maximum late at night. The water level in the eastern observation well (OW2) decreased distinctly more during water extraction than the water level in the western observation well (OW1) (Fig. 5). Consequently, the water gradient increased during extraction from west to east, i.e. from the regional groundwater towards the river, and therefore towards the extraction well. The latter means that the contribution of regional groundwater increased in the extraction well during extraction periods. Average water levels for both the daily minima and maxima showed slight increases on days 2, 5, and 10 (Fig. 5), corresponding with the precipitation events and resultant increases in TCC values in the extraction well on the same days (Fig. 2). This clearly demonstrates that the precipitation-induced increase of the river stage was transferred to the riverine groundwater and propagated further into the aquifer. Moreover, during high-discharge events the difference in water levels (Fig. 5) was slightly negative (i.e. more river water infiltrate flowing towards the extraction well) during non-extraction periods and considerably smaller during extraction periods (particularly during Event 1).

## Discussion

The high-frequency flow cytometry data revealed both an aperiodic and a periodic (diurnal) dynamic in bacterial concentrations in the groundwater extraction well (Fig. 2B). The aperiodic dynamics corresponded with precipitation events and related river stage fluctuations (Fig. 2A), whereas water extraction data (Fig. 2C) and the local groundwater levels (Fig. 5) explained the periodic, diurnal fluctuations. In this particular example, bacterial concentrations in the groundwater extraction well were influenced simultaneously by natural and operational events.

### Natural events influence bacterial cell concentrations in groundwater

TCC in groundwater is typically low (ca. 10^1^ cells μL^−1^) compared to surface waters (ca. 10^3^ cells μL^−1^)^41^,^42^. The TCC values in the extracted groundwater from the present study were high for groundwater (between 0.9–3 × 10^2^ cells μL^−1^), which is attributed to the close proximity of the extraction well to the river with a consequently short retention time of bank-filtered water in the subsurface. The time difference between the peak in the river stage (Fig. 2A), and the TCC peak in in the extraction well (Fig. 2B) was approximately 15 hours. This is a rather short travelling time for groundwater, considering legal guidelines in a number of countries that require travelling times of 10–50 days and more^43^. Precipitation-induced high-discharge events during the sampling period resulted in increased TCC values in the groundwater in the extraction well (Fig. 2A and B), which is primarily attributed to higher TCC in the infiltrating river water. Previous studies showed that precipitation causes considerable increases in bacterial concentrations in river water^24^,^34^,^44^,^45^, where the dilution effect of the rainwater is substantially outweighed by bacterial inputs from surface run-off, agricultural run-off, wastewater and storm water overflows, and suspension of sediments^46^,^47^, For example, Besmer *et al*.^34^ showed TCC values in river water more than doubling even after a small precipitation event.

In addition to the higher concentrations of bacteria in the river water, the higher river water stage amplified the effect with respect to increased TCC in groundwater in the extraction well. The differences in groundwater levels in the observation wells (Fig. 5) indicate that during Event 1, the relative amount of infiltrating river water was higher both during pumping and non-pumping conditions compared to their equivalents after the high-discharge event. In summary, during (large) high-discharge events, the groundwater in the extraction well receives more infiltrated river water with a substantially elevated TCC. This combination of amplifying effects probably also explains the above-described large fluctuations on days 2 and 3 that could not be explained adequately by the time series decomposition (Fig. 3A). These findings add to previous investigations of the influence of hydrogeological conditions on bacterial dynamics by moving from observations at seasonal time scales to short-term dynamics^17^,^30^.

While an increase in TCC has no direct hygienic implication *per se*, it is well documented that sources of fecal contamination (e.g., agricultural run-off, wastewater treatment plant discharge, soil particles) are released into rivers during/after precipitation events^44^,^48^,^49^. Moreover, Page *et al*.^24^ previously showed breakthrough of indicator bacteria in riverbank-filtered water following precipitation events and the vulnerability of groundwater systems to precipitation is generally recognized^11^,^16^,^42^. In the present study we did not measure specific indicator bacteria (e.g., plating for *E. coli*) or microbial community shifts (e.g., 16S sequencing). The focus in this study was on detection of events at high temporal resolution, which are not feasible for these more specific methods given current sampling/measuring limitations. Nonetheless, the flow cytometric fingerprint data suggests measurable shifts in percentage of LNA content bacteria after precipitation events (Fig. 4). The increase in both the percentage and absolute abundance of LNA content bacteria after precipitation events is interesting. Previous studies have shown that changes in FCM fingerprints are typically associated with changes in community composition^50^,^51^,^52^. However, a previous study suggested that HNA content bacteria (rather than LNA content bacteria) increased in river water after precipitation events^34^. The river water was not measured in the present study, and it can therefore not be excluded that a community shift towards more LNA content bacteria occurred in the raw water. In addition, the subsurface filters out infiltrating river water bacteria and at the same time allows detachment of indigenous bacteria from biofilms present in the subsurface. Changes in these latter two processes during increased river water influx may contribute to the shifts in the relative abundance of LNA content bacteria in the extraction well. The present data set is inconclusive about the exact cause of the FCM fingerprint shifts, for which follow-up work with source tracking and in-depth community composition analysis would be required. Given the dynamic nature of the precipitation events, a rapid, quantitative and online descriptive measurement of the microbial dynamics such as TCC and FCM fingerprinting can be highly valuable as an early warning system and for more advanced analysis (see below).

### Operational practices impact bacterial cell concentrations in groundwater

The idea that operational practices can influence bacterial concentrations in engineered systems is not new. Already in 1894, Frankland and Frankland described how water extraction from wells after stagnant periods resulted in increased numbers of bacteria detected with heterotrophic plate counts^53^. Today, flow cytometric TCC is available as a more meaningful process variable in drinking water treatment and distribution. For example, Vital, *et al*.^54^ characterized microbiological changes during drinking water treatment steps such as ozonation and biological filtration with flow cytometry, while Gillespie *et al*.^55^ assessed the effect of (residual) chlorine on bacteria during drinking water distribution. In the present study, a periodic, diurnal pattern observed in TCC and, less prominently, in the flow cytometric fingerprints (Fig. 4), is another clear example of the detection of operationally induced dynamics in bacterial abundance and composition with flow cytometry. While Page *et al*.^27^ found diurnal patterns in abiotic parameters in comparable settings of riverbank filtration and intermittent water extraction, to our knowledge nothing is known until now about the influence of regular but intermittent groundwater extraction on microbial dynamics.

From the temporal coincidence of the diurnal decreases in TCC (Fig. 2B) and the operation intervals of the extraction pumps (Fig. 2C), we infer that water extraction lowered the TCC of the water in the extraction well. This can be explained based on the hydrogeological setting (Fig. 1) and the change in hydraulic gradient during extraction (Fig. 5). The water in and around the extraction well is a mixture of two groundwater components, i.e. regional groundwater and locally infiltrated river water. At the point of extraction, the regional groundwater component is characterized by water that has a comparably long residence time in the subsurface (in the orders of days), whereas the locally infiltrated river water only has a residence time of several hours. The intermittent extraction of groundwater changes the local groundwater levels and thus also the gradients that determine the flow in the aquifer (Fig. 5). Although no samples from the observation wells were available, we here argue that the regional groundwater had lower TCC values than the locally infiltrated river water, and therefore the shift in mixing ratios discussed above would account for the TCC fluctuations observed in the extraction well. In this study, extraction led to lower TCC values that are indicative of better/longer filtered groundwater. However, in a different hydrogeological setting the effect could well be the opposite, i.e. extraction may for example increase the share of local river water infiltrate and thus lower the quality of the extracted water. In an extreme case, new aquifer compartments might start to contribute groundwater due to the extraction^56^ and thus potentially influence the (microbial) water quality (through related agricultural sources, landfills, deeper aquifers, etc.)^57^.

### Microbial baselines can be dynamic

Due to the dynamic nature of many natural and engineered aquatic systems used for drinking water production, fluctuations in different variables often occur on regular scale in absence of any dramatic system disturbance^58^,^59^. We previously detected diurnal patterns in the TCC of river water and in continuously flowing drinking water in a building plumbing system^34^. Similar to those studies, the extraction-driven diurnal pattern in the present study (Fig. 2B) challenges the concept of a static TCC baseline representing standard conditions of a system, and of any deviation from that line indicating a system disturbance. In this example, the TCC baseline is dynamic in time and consists of two basic system states (i.e. groundwater extraction and no groundwater extraction) with two main, time-dependent TCC levels. Thus, aperiodic dynamics – such as those observed following precipitation events and subsequent river stage increases – have to be evaluated against time-dependent baseline values (derived from the periodic dynamic) reflecting the operational situation.

The combined “trend” and “seasonality” components in Fig. 3A explain the vast majority of TCC measurements well. Based on this, the extracted “seasonality component” is a good descriptor of the dynamic baseline against which the other measurements can be compared (Fig. 3B). While the temporal evolution and the magnitude are clearly supported by the data, the exact concentrations for this baseline would require further investigations, including the impact of seasonal changes. It is clear that the observation period of 14 days was not long enough to record enough baseline data for a complete characterization of this specific system. The system probably did not reach real steady-state conditions during the observation period, which is evident when compared with the dry weather TCC values in Figure S1 for example. Nevertheless, this example illustrates the potential for real-time system monitoring and sophisticated event detection in the presence of a dynamic baseline due to alternating system states^60^.

### Potential applications and consequences of high frequency microbial data

Water utilities are responsible for ensuring high quality drinking water. To achieve this, operators and managers of water treatment plants require accurate information about raw water quality, treatment efficacy, and the stability of the treated water^61^. Knowledge of the variability of microbial loads and peak concentrations in raw water for drinking water treatment allows for better risk assessment and management of the treatment (e.g., disinfection dosage or water discarding alarms)^61^. When the influence of operational practices (e.g., intermittent extraction, filter -backwashing) is quantified in detail, it elucidates and explains average microbial concentrations measured in reservoirs or distribution networks (see the work of Kistenmann *et al*.^62^ on peak loads in raw water reservoirs and of Besner *et al*.^63^ on intrusion events in distribution networks). Furthermore, it allows for efficient tailor-made measures to hedge against the risk of breakthroughs in biological filters^61^ or to avoid the input of peak loads into the distribution system (which can be much easier and/or cheaper than trying to decrease average loads). Moreover, relevant proxy variables (e.g., turbidity, electrical conductivity, pH, SAC_254_) may be found that are easier/cheaper to measure than TCC, and which correlate sufficiently with bacterial concentrations^36^,^64^.

Routine monitoring and quality control by water utilities and/or legal authorities can be optimized based on detailed knowledge of dynamic baselines and a system’s response to specific events. Representative sampling time points can be identified in order to optimize the sampling frequency (and thus labor and cost)^65^. This would enable strategic application of complementary analytical methods on critical time points (e.g., indicator cultivation, general or target-specific sequencing, ATP measurements). In this specific example, some of the major changes in water quality occurred at night, when routine measurements are not commonly carried out. Based on the automated online flow cytometry measurements, these changes were identified and can now be tested specifically to assess their consequences for water quality. In this regard, one approach may be automatically triggered sampling for more specific measurements when increasing trends of TCC are detected, i.e. smart sampling^66^. In addition, any future (grab sampling) measurements can be evaluated against the dynamic baseline and thus interpreted more meaningfully.

With all sorts of (abiotic) automated, online measurements implemented in water quality monitoring^7^,^27^, the introduction of microbial online tools seems the logic next step^34^,^35^,^67^. This would allow for real-time quality monitoring with automated alerts and even operational measures (e.g., discarding of water, additional treatment, higher disinfection doses) in the case of relevant deviations from established (dynamic) baseline conditions. It also offers considerable potential for combined abiotic and microbial measurement systems with complementary information on different water characteristics^68^. Major remaining challenges are automated and meaningful data processing, as well as developing online systems that are financially feasible for small/medium scale water utilities as well.

## Additional Information

**How to cite this article**: Besmer, M. D. *et al*. Online flow cytometry reveals microbial dynamics influenced by concurrent natural and operational events in groundwater used for drinking water treatment. *Sci. Rep.* **6**, 38462; doi: 10.1038/srep38462 (2016).

**Publisher’s note:** Springer Nature remains neutral with regard to jurisdictional claims in published maps and institutional affiliations.

## Supplementary Material

Supplementary Information
