# Supplementary material for: Online flow cytometry reveals microbial dynamics influenced by concurrent natural and operational events in groundwater used for drinking water treatment
Source: Sci Rep. 2016 Dec 7;6:38462. doi: 10.1038/srep38462 (PMC5141442; doi:10.1038/srep38462)
Supplement: Supplementary Information [file srep38462-s1.pdf]

Supplementary information:

Online flow cytometry reveals microbial dynamics influenced by concurrent natural and operational events in groundwater used for drinking water treatment

Michael D. Besmer, Jannis Epting, Rebecca M. Page, Jürg A. Sigrist, Peter Huggenberger, Frederik Hammes

Contents:

1. Flow cytometry measurements of preliminary sampling campaign
2. Daily maxima and minima in total cell concentration
3. Differences in total cell concentration between extraction and non-extraction periods

1. Flow cytometry measurements of preliminary sampling campaign

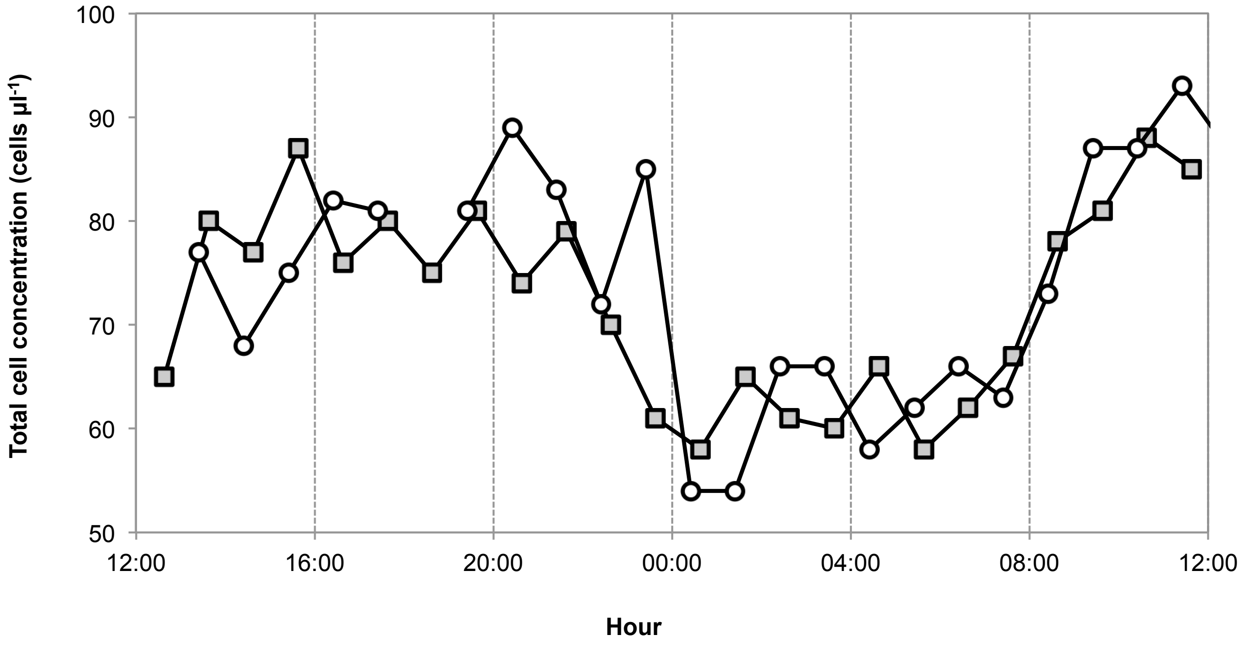

Figure S1: Flow cytometric total cell concentrations (TCC) from the extraction well (Figure 1, main manuscript) collected during a dry weather period every hour with an auto-sampler (Sigma) during a preliminary 48-h-sampling campaign. The data show TCC values fluctuating between 54 – 93 cells ul^{-1} (average: 73 cells ul^{-1}), with a seemingly diurnal pattern in the data.

2. Daily maxima and minima in total cell concentrations

Table S1: Average maxima and minima of total cell concentrations (TCC) from Figure 2B (main manuscript). For a straightforward overview of the diurnal fluctuations in TCC, a moving average covering 9 data points (approximately 105 min) was used and the daily minimum and maximum values displayed.

Day	**Maximum TCC** (cells μl^{-1})	**Minimum TCC** (cells μl^{-1})	**Maximum %LNA** (%)	**Minimum %LNA** (%)
01	171.0	127.2	78.1	77.1
02	271.8	122.5	79.3	77.5
03	237.8	166.6	80.4	76.8
04	175.3	133.4	83.4	80.7
05	161.5	121.7	81.7	78.5
06	176.3	122.3	79.5	77.8
07	159.5	118.3	79.7	77.5
08	152.4	114.6	77.8	75.8
09	134.0	106.3	76.4	74.7
10	135.3	100.9	75.8	74.2
11	139.5	105.4	76.0	74.2
12	138.8	103.1	76.7	74.6
13	130.0	102.6	77.4	75.3
14	124.6	97.7	76.3	74.6

3. Differences in total cell concentration between extraction and non-extraction periods

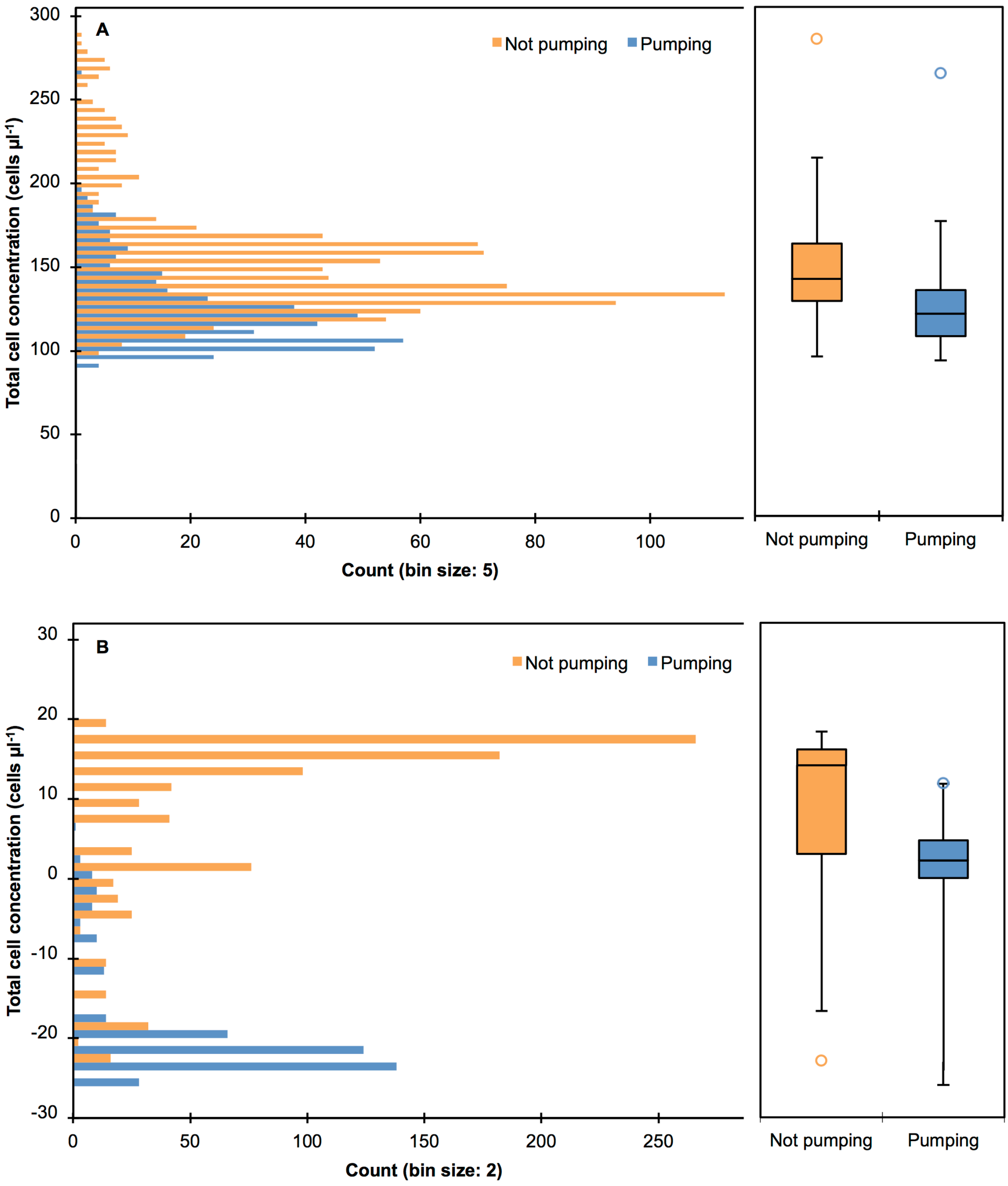

Figure S2: Histograms and box plots of the differences in TCC levels during extraction (blue) and non-extraction (orange) periods. Panel A contains the entire data set (from Figure 2B; *n = 944*) and panel B contains the data set of the "seasonal component" (from Figure 3A; *n = 443*). The concurrent precipitation event partly masked the differences in panel A. For both evaluation, the short transition periods between extraction and non-extraction periods, in which TCC changes, masked the differences slightly.
